# Exotic self-assembly of hard spheres in a morphometric solvent

**DOI:** 10.1073/pnas.2314959121

**Published:** 2024-04-04

**Authors:** Ivan Spirandelli, Rhoslyn Coles, Gero Friesecke, Myfanwy E. Evans

**Affiliations:** ^a^Institute for Mathematics, University of Potsdam, Potsdam 14476, Germany; ^b^Institute for Mathematics, Technical University Berlin, Berlin 10623, Germany; ^c^Faculty of Mathematics, Technical University Chemnitz, Chemnitz 09107, Germany; ^d^Department of Mathematics, Technische Universität München, Garching 85748, Germany

**Keywords:** self-assembly, hard sphere clusters, morphometric model, geometry, colloids

## Abstract

The self-assembly of spheres into geometric structures, under various theoretical conditions, offers valuable insights into complex self-assembly processes in soft systems. Previous studies have utilized pair potentials between spheres to assemble maximum contact clusters in simulations and experiments. The morphometric approach to solvation free energy that we utilize here goes beyond pair potentials; it is a geometry-based theory that incorporates a weighted combination of geometric measures over the solvent accessible surface for solute configurations in a solvent. In this paper, we demonstrate that employing the morphometric model of solvation free energy in simulating the self-assembly of sphere clusters results, under most conditions, in the previously observed maximum contact clusters. Under other conditions, it unveils an assortment of extraordinary sphere configurations, such as double helices and rhombohedra. These exotic structures arise specifically under conditions where the interactions take multibody potentials into account. This investigation establishes a foundation for comprehending the diverse range of geometric forms in self-assembled structures, emphasizing the significance of the morphometric approach in this context.

## Significance

Microscopic soft structures, such as biomolecules, colloids or proteins self-assemble into a plethora of shapes, some of which are both complicated and spectacular. The mechanisms behind how these structures assemble into these shapes, and change form under different conditions, provide insight into structure and function. This paper provides a theoretical setting under which to simulate the self-assembly of sphere clusters in a solvent, where the resulting clusters form exotic shapes such as double helices and isohedral rhombohedra. This demonstrates that complicated geometry can stem from simple physical conditions, improving our understanding of the microscopic world.

Self assembly is an important process in which intricate structures with particular shape and function form based on microscale energetic considerations, such as in colloid self-assembly or protein folding. At the heart of any such self-assembly process is a local interaction between building blocks dictating a specific global arrangement. An intriguing breadth of geometric structures result from subtle differences in physical conditions and interactions.

Analyzing hard sphere systems is a way to isolate the contribution that liquid properties and particle-liquid interactions make to the self-assembly process in a controlled way. When the hard sphere systems are endowed with a short ranged pair-wise interaction, the systems are somewhat binary: They can maximize the pairwise contacts between particles, or they can dissociate or dissolve. In previous studies, maximum contact clusters are shown to be ground states for short ranged attractive potentials (1), as well as minimizing the second moment (2) and some Lennard-Jones clusters exhibit equivalent topology (3). Experimentally, maximum contact clusters are observed as results of colloidal cluster formation driven by depletion forces (4).

In this paper, we consider solvation effects on hard sphere clusters using a geometry-based technique that accounts for multibody interactions between particles: the so-called morphometric approach to solvation free energy (5, 6). The morphometric approach offers a highly efficient and accurate method for calculating the solvation free energy of solutes with complex shapes. It is founded on Hadwigers theorem of integral geometry (7, 8), which allows us to write an additive, motion invariant and continuous functional ϕ of a union of convex bodies K as a linear combination of only four geometric measures of K. These measures are volume V, surface area A, integrated mean curvature C and integrated Gauss curvature X. The integrated Gauss curvature equals 2π times the Euler characteristic. The calculation then utilizes these four geometric quantities computed over the solvent accessible surface around the solute, along with thermodynamic prefactors determined by the solvent properties.

The morphometric approach builds on a multitude of previous ideas, which introduce a subset of the geometric terms. The first is the definition of the solvent-accessible surface (9), defined as the space filling diagram of the molecule, inflated by the radius of water. This concept is subsequently extended to a model of solvation free energy as a sum of surface effects, weighted by the per atom contributions to the solvent accessible surface (10, 11). It was later found that, for small solutes, the solvation free energy can be better related to the excluded volume of the molecules (12, 13).

The morphometric approach to solvation free energy has been used to accurately calculate the solvation thermodynamics of proteins and ligands (5, 14, 15) and the thermodynamic potential of hard sphere fluids depending on the shape of the container (6). It has been used to compute the solvation properties of skin cells (corneocytes), whose microstructures display complicated geometry (16). More recently, the morphometric approach was derived as the exact resummation of terms in the virial series (17), further justifying the approach.

**Fig. 1.**
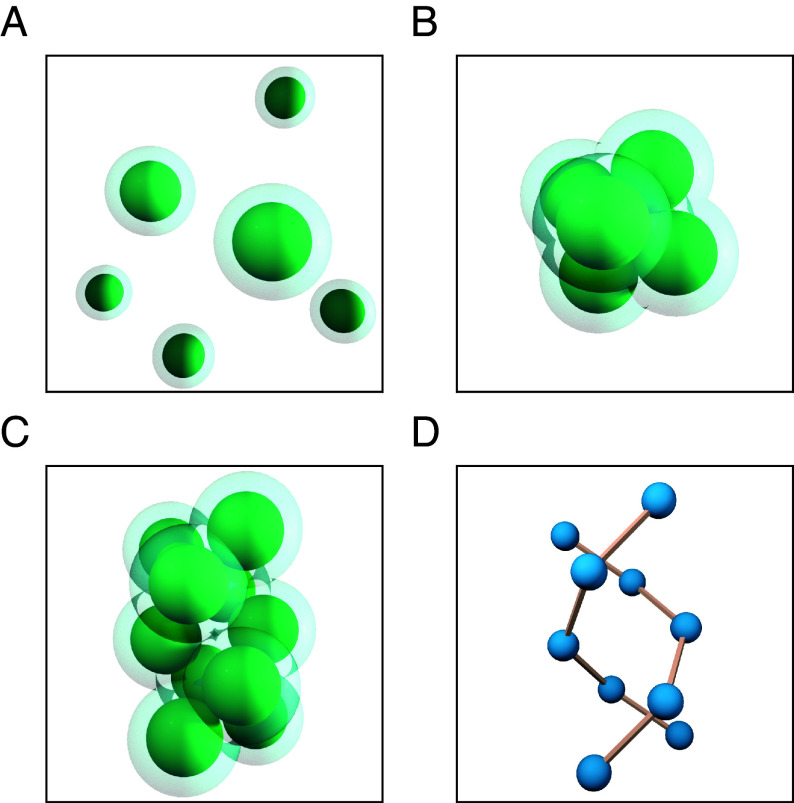
Hard spheres are shown in green with overlapping, transparent surfaces around them representing the solvent accessible surface. (*A*) Six disjoint particles in a dissolved state. (*B*) Six particles in a maximum contact cluster (1, 4, 18). (*C*) An exotic configuration found as a minimizer in simulations: ten particles in a double helix. (*D*) The contact graph of hard spheres for the double helix, with sphere centers depicted as spheres and sphere contacts depicted as edges.

In the present context, the union of convex bodies K represents spheres (a solute) immersed in the solvent, which is modeled implicitly by inflating the spheres by a distance equal to the radius of the solvent particles. See Fig. 1 for an illustration. The morphometric approach tells us that the solvation free energy of the solute particles in the solvent takes the form:

[1] $$\begin{array}{c} F_{\mathrm{sol}}=pV+\sigma A+\kappa C+\bar{\kappa }X, \end{array}$$

with solvent pressure p, surface tension σ and bending rigidities κ and $\bar{\kappa }$, being constants depending on the solvent properties in the thermodynamic system. We get the thermodynamic coefficients from the white bear functional of fundamental measure theory (19), which gives us p, σ, κ, and $\bar{\kappa }$ depending on the radius of the solvent $r_{s}$ and the solvents packing fraction η. Further discussion of these thermodynamic parameters is in Section 2.

This paper implements the morphometric approach to solvation free energy in a simulation setting, where a physical simulation gets converted to a geometric one, with the main computational considerations being the efficient computation of the geometric quantities of the solute configurations. We do this on finite clusters of hard spheres immersed in a solvent with varying properties, finding the configuration which minimizes the energy over all possible configurations of the spheres using a simulated annealing algorithm (20) based on the Metropolis algorithm (21) (details are provided in the *Materials and Methods*). The advantage of the morphometric approach is that the main computations performed in the simulation are purely geometric and we utilize efficient techniques from computational geometry (22–26). The minimizers that we obtain for different solvent properties are an interesting array of both expected maximum contact clusters and new exotic configurations, which we detail below.

## 1. Results

We investigated the minimal energy configurations of clusters of hard spheres in various solvents, as a function of solvent radius and packing fraction. These configurations are found through simulated annealing as well as further comparison of exact computations over all configurations. The parameter space that is explored is the ratio of solvent to solute radius $\frac{r_{s}}{R}\in \left(0.0,0.5\right)$ and solvent packing fraction η∈(0.0,0.494), always taking a repulsive surface interaction of the spheres. The upper limit of 0.494 for η corresponds to the value at which the hard sphere fluid freezes. Accurate prefactors for higher packing fractions have recently been developed in ref. 27. The exploration of these parameters changes the thermodynamic prefactors in the calculation of the solvation free energy, which simply changes the relative weighting of each of the geometric terms. If we consider a fixed value for $r_{s}$ and R and a fixed sphere configuration, the minimizer depends only on the weighting of those measures, which is given by the prefactors $p,\sigma ,\kappa ,\bar{\kappa }$.

Some general observations about the prefactors in our parameter space are that $p>|\sigma |>\kappa >|\bar{\kappa }|$, where the magnitude of each coefficient is approximately less than the previous one by an order of magnitude. Furthermore p is always positive, which implies that volume minimizing structures are favored. The sign of σ is always negative however, which favors area maximizing structures. The mean curvature prefactor κ is always positive, implying mean curvature minimizing structures are favored. Finally, $\bar{\kappa }$ is very small and has negative sign across the parameter space.

We characterize a cluster of hard spheres by the graph of contacts between the hard spheres, so each sphere is represented by a vertex and a contact by an edge. Contact detection is done with a tolerance of $2R+\frac{r_{s}}{2R}$, including spheres that are very close to touching. In general, it is possible for the solvent accessible surfaces around spheres to be “interacting” without a contact between the spheres themselves.

**Fig. 2.**
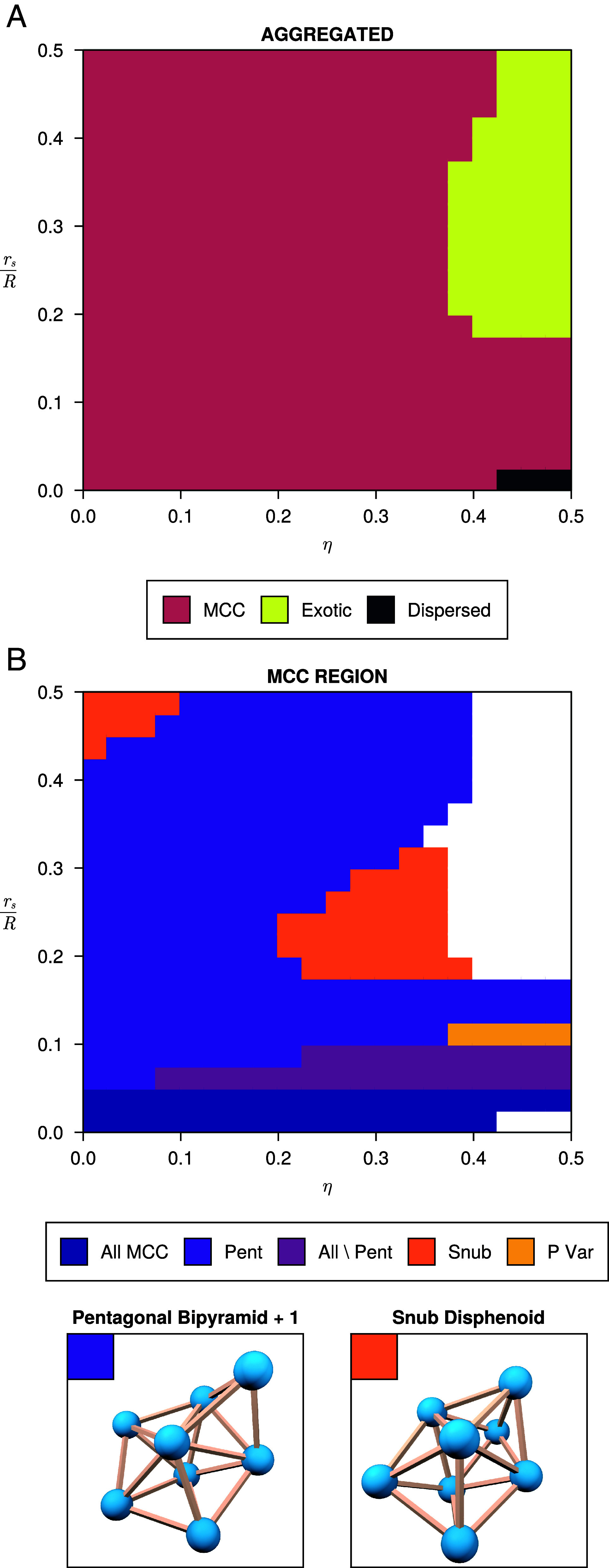
(*A*) Aggregated phase diagram of the self-assembly process, including the region of maximum contact clusters (MCC), exotic clusters and the disperse phase for n=4,⋯,8. We aggregate the results by marking a region as a particular type if it forms for a majority of n. (*B*) Phase diagrams of the maximum contact cluster region for n=8 (excluding the exotic region shown here in white). The region marked “All MCC” has all thirteen MCC for n=8 as minimizer. “Pent” and “Snub” refer to the pentagonal bipyramid with and added ball and the snub disphenoid. “All \ Pent” is minimized by all MCC except “Pent.” “P Var” refers to three different clusters that are similar to “Pent” but the *Top* and *Bottom* of the pyramid touch while there is a gap in the pentagonal ring.

We perform simulations of the minimization of 4, 5, 6, 7 or 8 spheres in a simulation space for sets of coefficients in the parameter space. We obtain minimizing configurations for each combination of variables, resulting in the phase diagrams which are shown in Fig. 2.

Across all choices for the number of spheres in the clusters, we see similar behavior. Given the similarity of the phase diagrams for different numbers of spheres, we make an aggregated diagram for all numbers of spheres, where regions are colored based on which phase occurs more often. This generic diagram is shown in Fig. 2*A*. A large region of the phase diagram are maximum contact clusters, which is consistent with previous simulations involving short ranged pairwise potentials. Maximum contact clusters maximize the number of contacts between spheres and have recently been enumerated for n≤19 (18, 28), with their optimized coordinates maintained as a dataset (29). We will discuss these maximal contact clusters in Subsection 1.1.

A phase consisting of dispersed spheres occurs in a very small corner of the diagram. We call a cluster dispersed if it has no hard sphere contacts. These configurations may, however, have interactions between the solvent accessible surfaces of nearby spheres. Analytically, we find that the optimal configurations in this small region have just touching inflated particles, as discussed later in Section 2.

The final region we consider includes what we term exotic clusters, which show behavior that are neither maximal contact clusters nor dispersed, such as the double helical structure shown in Fig. 1. There are a wide variety of interesting geometric conformations that occur as minimizers in this region, and we will present these structures in detail in Subsection 1.2.

### 1.1. Maximum Contact Clusters.

For sufficiently small $r_{s}$, $F_{\mathrm{sol}}$ acts as a pairwise short ranged attractive potential. To see this consider three pairwise touching hard spheres with radius R. Their centers are arranged in an equilateral triangle with edge length 2R. The circumscribing radius of the equilateral triangle is given by $\frac{2R}{\sqrt{3}}$. Therefore, for $r_{s}\geq \frac{2R}{\sqrt{3}}-R$, the inflated balls have common intersection. Meaning for $\frac{r_{s}}{R}<\frac{2}{\sqrt{3}}-1$, the energy functional $F_{\mathrm{sol}}$ describes pairwise interactions of the particles. Within this regime, $F_{\mathrm{sol}}$ will scale with the number of contacts in the cluster. The maximum contact clusters are the set of clusters that maximize the contacts between spheres, and so we get that all of the maximum contact clusters minimize $F_{\mathrm{sol}}$ for small solvent radii $\frac{r_{s}}{R}$, with all clusters having an equivalent $F_{\mathrm{sol}}$. Our experiments and subsequent analytical calculations suggest that some particular maximum contact clusters minimize $F_{\mathrm{sol}}$ for a large part of the phase diagram, even beyond the pairwise interactions. Note that the area of the phase diagram where maximum contact clusters are minimizers matches the region where minimizing volume dominates the energy.

A phase diagram of the different regimes in the maximum contact cluster region for n=8 is shown in Fig. 2*B*. For n=8, there are a total of thirteen possible maximum contact clusters, including the pentagonal bipyramid with an added ball and the snub disphenoid, both shown below the phase diagram in Fig. 2*B*. As expected for small solvent radii, there is a region in which all maximum contact clusters have the same solvation free energy. This region extends up to $\frac{r_{s}}{R}\approx 0.05146$, which is smaller than the pairwise interaction threshold calculated above. As $\frac{r_{s}}{R}$ exceeds this threshold, the phase diagrams splits into two regions. One where only the pentagonal bipyramid with an added ball minimizes $F_{\mathrm{sol}}$ (purple) and one where all other maximum contact clusters have the same energy (wine red). This transition is caused by the fact that at $\frac{r_{s}}{R}\approx 0.05146$ the top and bottom of the pentagonal bipyramid start to interact, which is either favorable or unfavorable depending on η. The pentagonal bipyramid with an added ball is a minimizer over a large region of the phase diagram. The snub disphenoid is also a minimizer for some regions of the phase diagram, which is the most stable Lennard-Jones cluster at n=8 (3).

Although the groups or individual clusters minimizing $F_{\mathrm{sol}}$ vary across the parameter space, the geometric measures of all maximum contact clusters are typically very similar. Accordingly their solvation free energy values are too, which gives us a free energy landscape with several local minima close to the global minimum that are all comparatively close in depth.

### 1.2. Exotic Clusters.

**Fig. 3.**
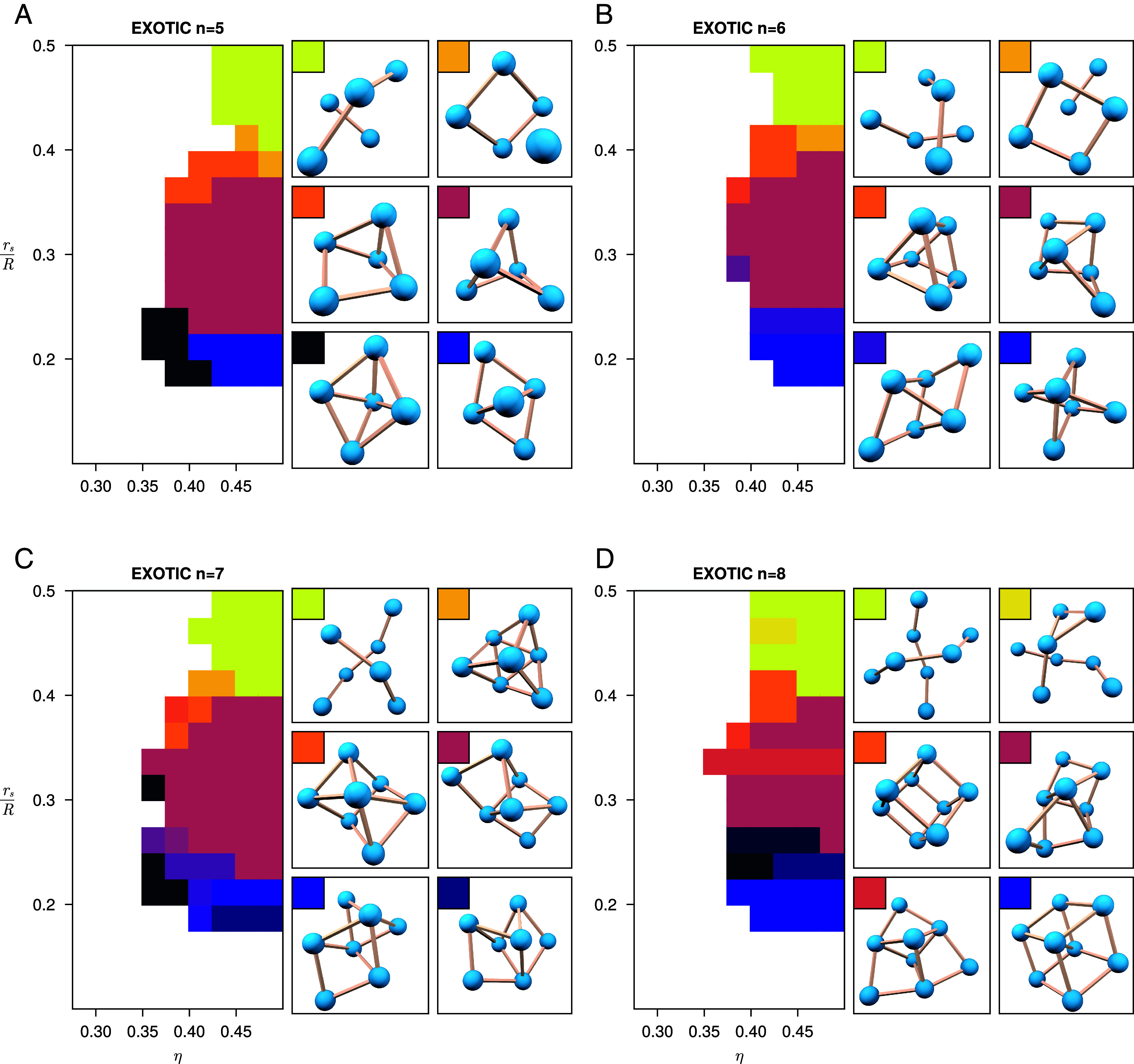
Phase diagrams of the region of exotic clusters for (*A*) 5 spheres, (*B*) 6 spheres, (*C*) 7 spheres and (*D*) 8 spheres, where η is the solvent density and $\frac{r_{s}}{R}$ is the sphere radius relative to the solvent radius. Different minimizing structures are represented by different colors in the phase diagrams, and a selection of these clusters are shown beside each phase diagram together with their color from the phase diagram. Of particular note are the bright yellow structures on the *Top Left* of each set of images, as these are a sequence of double helices of increasing length.

A variety of so-called exotic clusters are minimizing structures when the solvent density is high. The contact graphs have edges denoting spheres that are in contact (with the same tolerance $2R+\frac{r_{s}}{2R}$). In exotic clusters, the interactions between the solvent accessible surfaces of spheres that are not in contact lead to interesting geometric formations. The regions of the phase diagram where different exotic clusters are the minimizers are shown in Fig. 3 for clusters of spheres containing 5, 6, 7, and 8 spheres. Clusters are grouped by isomorphic contact graphs. Furthermore we sometimes split up groups of clusters with isomorphic contact graphs if the clusters are substantially different in terms of geometry, in particular with angles between neighboring edges.

**Fig. 4.**
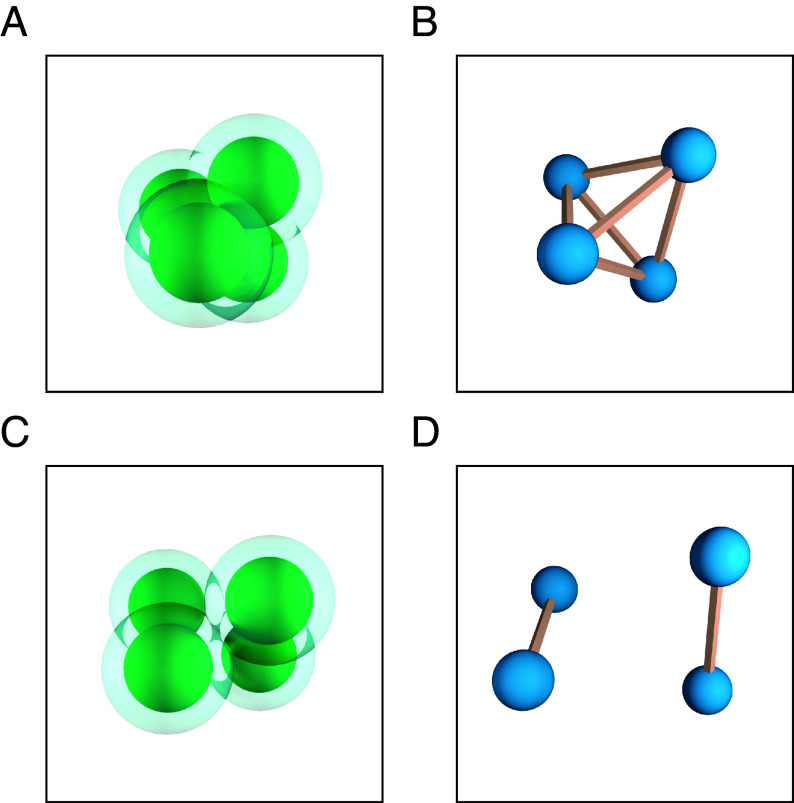
Cluster (*A*) and contact graph (*B*) of a tetrahedron, as well as cluster (*C*) and contact graph (*D*) of a cluster of a four sphere configuration found to be a minimizer at $\frac{r_{s}}{R}=\eta =0.475$.

We begin with the example of four spheres, not pictured in the Figure. We take a specific set of parameters, namely $\frac{r_{s}}{R}=0.475$, η=0.475. If $F_{\mathrm{sol}}$ were only taking pairwise interactions into account, the minimizing configuration would be a tetrahedron of edge length 2R, which is the maximum contact cluster. The minimizing cluster we found for these parameters has two pairs of spheres in contact, and the interaction of the solvent accessible surfaces between the pairs holds them in close proximity to each other. See Fig. 4 for an illustration of the respective clusters and contact graphs. This simple example shows that energy considerations beyond pairwise interactions modeled by $F_{\mathrm{sol}}$ are essential to the formation of exotic clusters.

As we move to larger numbers of spheres, the minimizing clusters become more diverse, with varying symmetry and graph connectivity. For clusters of five spheres, we see six main exotic clusters arising, all pictured in Fig. 3*A*. One of these structures is a precursor to a double helix, with two chains of length two and three respectively interacting along their length. Another structure contains a single sphere suspended above a square, and some interesting structures with 3-fold and 4-fold symmetry also form. When we move to clusters of six spheres (Fig. 3*B*), we get more variety. The double helical structure stays, although now balanced with three spheres in each chain. We also get a symmetric triangular prism and a structure with 4-fold symmetry.

**Fig. 5.**
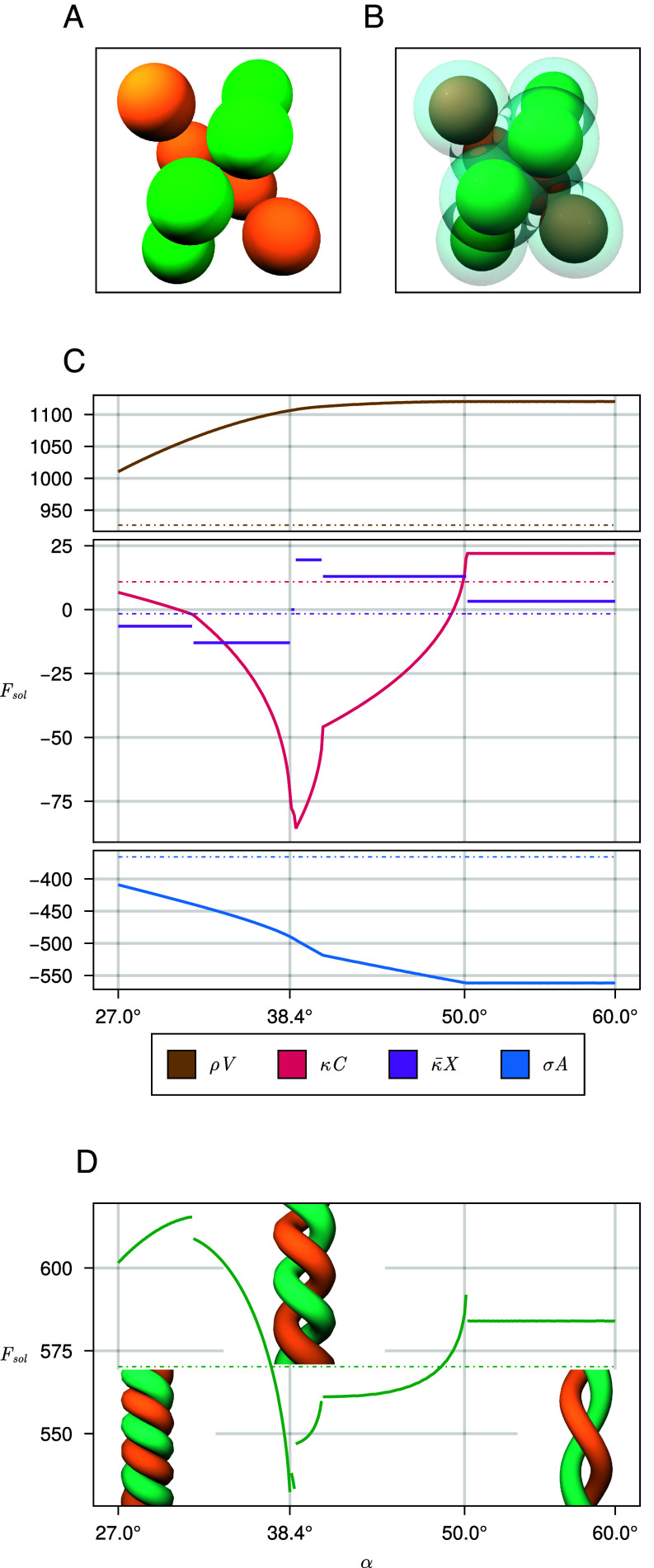
(*A*) Hard spheres of a double helix configuration, found as a minimizing structure for a particular region of the phase diagram. (*B*) The solvent accessible surface around the same double helix configuration. (*C*) The geometric measures weighted by prefactors for eight ball double helices with $\frac{r_{s}}{R}=\eta =0.475$ and helix radius 1.4R as a function of pitch angle α. We can see that the weighted volume increases with α, the weighted surface area decreases, and the weighted mean curvature has a deep minimum. The dotted lines represent the corresponding values for the snub disphenoid MCC. (*D*) Total $F_{\mathrm{sol}}$ as a function of α, with the dotted line represents the corresponding values for the snub disphenoid. The deep minimum of the weighted mean curvature leads to a deep energy minimum overall.

The number of exotic clusters found for 7 and 8 spheres is larger; we give a selection in Fig. 3 *C* and *D*. A remarkable configuration we found is that of the double helix. One of the minimizing helices composed of eight spheres is shown in Fig. 5*A*. The hard spheres of different strands are not in contact. This increases the volume the cluster excludes and the solvent accessible surface area, compared to similar configurations where the strands are closer together or in contact. The spheres of different strands inflated by the solvent radius $r_{s}$, do intersect however. Furthermore, the space between the strands features small cavities in the excluded volume. We also see the self-assembly of 10 spheres into a double helical configuration in our simulations, as shown in Fig. 1.

Dumbbells and axially symmetric discoids have previously been simulated and experimentally observed to self-assemble into helices (30, 31). For spherical colloids, this has been achieved experimentally (32–34) and in simulations, where the colloids are in cylindrical confinement (35). We report such simulated self-assembly for unconfined equally sized hard spheres. An animation of the simulation of eight spheres assembling into a double helix is given in *SI Appendix*. We explore their appearance as a minimizer in more detail here.

The parameter space where double helices are minimizers has the highest weights assigned to mean curvature minimization and area maximization. In general, as η gets larger, so does the impact of mean curvature minimization. Compared to the snub disphenoid S and the pentagonal bipyramid P, which both appear as minimizing clusters in the same region of $\frac{r_{s}}{R}$ just at lower solvent densities, the helix excludes more volume, which is energetically unfavorable, and has larger solvent accessible surface area, which is energetically favorable. If we only consider these two contributions to $F_{\mathrm{sol}}$, double helices would not be minimizer. However the helical configurations have negative mean curvature of large absolute value compared to small positive mean curvature of the other two clusters. Among the minimizers of the phase diagram for n=8, they have the highest negative mean curvature at the points in parameter space they minimize.

We calculate radius, pitch, and pitch angle parameterizations of double helices from the minimizing configurations. This is done by fitting a line through the midpoints of the four pairs of opposite hard spheres. Taking this line as the helix axis, we compute the average distance and angle between particles in the strand and their distance to the central line. The absolute value of ratio $\frac{P}{a}$ of helix pitch P and helix radius a range from 4.84 to 4.98. The helix radii, expressed in relation to solute radius R range from 1.34R to 1.42R. Each helix is constructed from approximately six hard spheres per turn. The pitch angles $\alpha =\;\text{atan}\left(\frac{P}{2\pi a}\right)$ lie between $37.6^{{}^{\circ}}$ and $38.5^{{}^{\circ}}$. The minimal $\frac{P}{a}$ and α increase with increasing $\frac{r_{s}}{R}$.

To analyze this further, we can generate hard sphere double helices of touching hard spheres whose centers lie exactly on double helical curves. With this, we generate a family of helices to analyze how the contribution of the different measures to the total energy change, depending on the pitch angle. Fig. 5 shows one such analysis for helix radius 1.4R and $\frac{r_{s}}{R}=\eta =0.475$. The minimizing helix we construct this way has a pitch of about $38.4^{{}^{\circ}}$, which fits the parametrizations of the helices found experimentally. In Fig. 5 *C* and *D*, we can see that the minimal helix is found in a region of high negative contribution of κC, skewed slightly by the negative Gaussian curvature contribution $\bar{\kappa }X$. This shows that both curvature terms play a role for the shape of the minimizing clusters, a significant motivation for the use of the morphometric approach. This is an idea that has been explored in the use of the morphometric approach in porous media (36). The discontinuities in the Gaussian curvature term correspond to topological changes of the configuration. Such topological changes could correspond to events such as the liquid being flushed from a cavity, for example, from the center of a helix that is shrinking. In practice, these discontinuities would be bridged by dynamics; however, we find the discontinuous behavior of topological invariants to be worth documenting in this case.

The formation of helices and other exotic clusters can be seen as a result of the counteracting forces of volume minimization on the one hand and surface area maximization plus mean curvature minimization on the other. This competition of energy terms is a form of frustration and has been observed to play an important role in the assembly of helicoids in settings in which a deformation energy counteracts an energy favoring aggregation of the assembling particles (37, 38).

In general, we observed that every minimizing exotic cluster has negative total mean curvature. This is also true for maximum contact clusters in certain ranges of $\frac{r_{s}}{R}$, but the absolute mean curvature values are generally smaller than those of the exotic clusters.

**Fig. 6.**
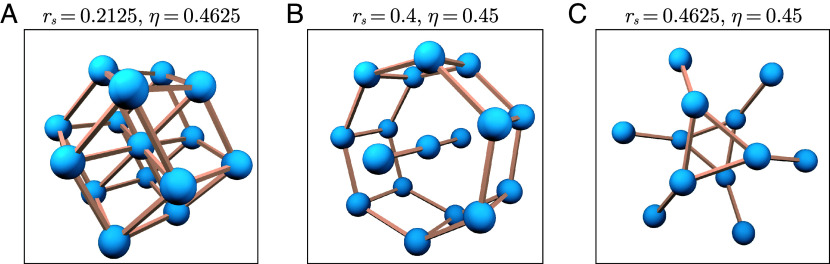
A selection of geometrically interesting minimizing configurations for n≫8 (*A*) 15 particles on a rhombohedral lattice forming a hull around a central particle whose geometry is a rhombic dodecahedron. (*B*) A coated string of hard spheres and (*C*) two interlocking hard sphere clasps. The parameters where these structures minimize are listed above the images.

One type of structure assembling for different cluster sizes has sphere centers on a rhombohedral lattice, as shown in Fig. 3 with dark purple color. These clusters form for 4≤n≤15, all within the same area of parameter space. The most recognizable cluster of this type is the isohedral rhombohedron for eight spheres. In the same area of the parameter space, we find a structure resembling a rhombic dodecahedron (with an additional sphere in the center) for n=15 which is displayed in Fig. 6*A*.

In Fig. 6 *B* and *C* we also show two other structures with interesting geometric form appearing for larger n. The first is a barrel shape with a stick through the middle, and the second is the interlocking of two star-like shapes with three-fold symmetry. In general for large n, we find a wide array of geometric forms as minimizers, surely of interest for future research.

## 2. Discussion

We have applied the morphometric approach to solvation free energy to find minimizing configurations of hard spheres. We use the simulated annealing algorithm to search for minimizing sphere clusters (details below in *Materials and Methods*). As with all simulation via a simulated annealing technique, we encounter the issues associated with the search for a global minimum versus local minima. We have countered this first through running significant simulation runs; roughly 800 simulation runs for each n spread across the parameter space. Further, we scanned the exotic regions for n∈{7,8} more thoroughly and ran 25 simulations for each point given by a combination of $\frac{r_{s}}{R}\in \left[0.0125,0.0375,0.0625,\cdots ,0.4875\right]$ and η∈[0.0125,0.0375,0.0625,⋯,0.4875], i.e., at each of the centers of the square regions in the phase diagrams. Finally, we ran an additional 75 simulations per point in parameter space that has a double helix as minimizer. Each simulation included at least five reheating steps further increasing the number of local minima attained during a simulation. The reheating process is explained in more detail in the materials and methods section. To increase the precision of the minimizers, we performed a further analytical comparison, where all possible configurations found in any part of the phase diagrams and the optimized mcc-configurations were tested as possible minimizers in other parts of the phase diagram. This process resulted in refinement of the phase diagram, in particular in the region of the mcc clusters.

We also compared double helices $H_{\mathrm{sim}}^{p}$ resulting from a simulation and minimizing $F_{\mathrm{sol}}$ at point in parameter space p, to double helices $H_{\mathrm{param}}^{p}$ generated lying on the parameterized curve with particle distance 2R. We found each pair to be close in terms of energy and pitch angle. The pitch angle of $H_{\mathrm{sim}}^{p}$ and $H_{\mathrm{param}}^{p}$ differ by at most $0.3^{{}^{\circ}}$ for all p. Furthermore for the corresponding energies $E_{\mathrm{sim}}^{p}$ and $E_{\mathrm{param}}^{p}$, we find that $0.95E_{\mathrm{param}}^{p}\leq E_{\mathrm{sim}}^{p}\leq 1.15E_{\mathrm{param}}^{p}$. The cases in which $E_{\mathrm{param}}^{p}$ is indeed larger than $E_{\mathrm{sim}}^{p}$ likely correspond to cases in which the true minimizing helix has the hard spheres at a distance slightly larger than 2R.

Given these measures of extensive simulation and analytical comparison, we are confident that our simulations are finding global minimizing structures for the morphometric solvation free energy, or at least very stable local minimizers. In real systems, there are additional free energies that contribute to self-assembly and stability of the clusters, such as the vibrational and permutational free energies (4). These free energies are not considered in the morphometric approach, and their inclusion would be a necessary extension to compute the probabilities with which the clusters assemble.

The tolerance introduced in identifying sphere contacts results in some transitional phases being aggregated together into a single structure. While this is on the one hand desired to group like structures, it also obscures some subtleties. There are some continuous deformations between minimizing clusters that are likely all minimizers, but these will be grouped into two distinct groups.

In the dispersed region of the phase diagram, the simulation finds dispersed spheres as the energy minimizer. In theory, a small decrease in energy is achieved by very slight overlaps of the solvent accessible surfaces. These slight interactions happen on occasion; however, the energy decrease is shallow which leads to the simulations mostly finding clusters in which there are no (or few) interactions between inflated balls. In theory, one could imagine a maximum contact cluster of slight interactions in the solvent accessible surfaces as being a minimizer, but this is never found computationally.

We established a broad overview of the parameter space of the solvation free energy of sphere clusters. We found maximum contact clusters as expected, extending the range over which these clusters are found, and identified a region where exotic clusters form. The span of parameters is large, and the most broadly relevant region of this parameter space lies toward the bottom, where the particles are large and the solvent small. Our results suggest that the maximum contact clusters will dominate, even when we move beyond a pair potential for small solutes. The exotic clusters are interesting, and we speculate that they might appear in mixtures of small and large colloids. Furthermore, we do not exclude that the conformations of the exotic region might also be found through other means, such as through coatings, gels, proteins, and other complex fluids, despite the fact that the theory of the morphometric approach relates specifically to hard sphere fluids. In particular, these structures may be stable cluster configurations in systems influenced by particle interactions and solvation effects. We also note that the exotic clusters are appearing in the vicinity of a predicted critical point for the demixing of binary hard sphere fluids.

Considerations of the symmetry of clusters go back almost a century (39). A general observation that has been made is that short range pair potentials disfavor symmetric clusters, while long range pair potentials favor them (4, 40, 41). The reason being that clusters forming under longer range pair-potentials can lower their energy by arranging noncontacting spheres in a symmetric way, such that the interactions are shared more equally. One study compares the energy landscapes and topologies of stable cluster sets of sticky hard spheres and Lennard-Jones potentials with different ranges (42). Among other things, it discusses how different clusters of 12 spheres touching one central sphere all optimize to an ideal icosahedral arrangement when a (6,12)-Lennard-Jones potential is applied. The morphometric approach models a short range potential; however, it also captures interactions of multiple spheres. It seems that to some extent a similar argument to that of longer range pair potentials can be made and indeed we see that several of the minimizing states we find exhibit symmetries.

Overall, this landscape of minimizing clusters provides insight into the complex relationship of geometry, solvation, and self-assembly. That a double helical chain of hard spheres should self-assemble under any conditions is a remarkable finding, and we hope that this leads to further understanding of self-assembly in general.

## 3. Materials and Methods

All minimizers are the result of a simulated annealing (20) algorithm used to decrease the solvation free energy ${\tilde{F}}_{\mathrm{sol}}$ of a randomly initialized distribution of n hard spheres. Here, ${\tilde{F}}_{\mathrm{sol}}$ = $F_{\mathrm{sol}}$ if the hard sphere cores of the particles do not overlap and ${\tilde{F}}_{\mathrm{sol}}=\infty$ otherwise. The coefficients defining $F_{\mathrm{sol}}$ are input parameters and remain constant throughout the optimization. At its core, our implementation is a single component Metropolis algorithm (21). Given a configuration $x^{i}$ of particle centers, we propose a configuration $x^{i+1}$ by randomly choosing one of the particles and translating it according to a normal distribution. We found that a good choice for the SD of the normal distribution is given by $\frac{r_{s}}{R}$, which means that the majority of translations perturb a particle within the range of $\frac{r_{s}}{R}$.

The decision of accepting $x^{i+1}$ as the new state of the simulation is done according to the Metropolis criterion. The state is accepted with probability

$$\;\text{min}\left(1,\;\text{exp}\left(\frac{-\Delta E}{T_{\mathrm{sim}}}\right)\right),$$

where $\Delta E=E\left(x^{i+1}\right)-E\left(x^{i}\right)$ is the difference in energy of the states. Note that $T_{\mathrm{sim}}$ is not the thermodynamic temperature but a simulation temperature. It regulates the probability of accepting energy increasing states. The energy is $E={\tilde{F}}_{\mathrm{sol}}$.

We employ a cooling scheme that decreases the simulation temperature $T_{\mathrm{sim}}$ with increasing number of iterations. As $T_{\mathrm{sim}}$ approaches zero, energy increasing deformations are extremely unlikely and the system converges to a local energy minimizing state. The temperature is initialized to $T_{0}$ and then temperature $T_{j}$ of simulation step j is set to

$$T_{j}=T_{0}{\left(\frac{J-j}{J}\right)}^{2},$$

where J is the total number of iterations. Extending the simulations by reheating several times after $T_{\mathrm{sim}}=0$ is reached causes more local minima to be attained, thus increasing the chance to find the global minimum. To find suitable initial temperatures, we implement an algorithm by Walid Ben-Ameur (43) that infers the temperature from a given set of Metropolis steps and a desired acceptance rate. For each of our simulations, we run one million Metropolis steps and vary the initialized acceptance rate between 0.5 and 0.65. Scripts containing computational instructions to reproduce our results can be accessed on GitHub (44).

The formulas for the dependence of the prefactors on the packing fraction are taken from the White Bear functional Mark II (19) and can be written as follows:

$$\begin{array}{c} \beta p=\frac{\eta }{r_{s}^{3}}\frac{3}{4\pi }\left(\frac{1+\eta +\eta^{2}-\eta^{3}}{{\left(1-\eta \right)}^{3}}\right), \end{array}$$

$$\begin{array}{c} \beta \sigma =\frac{\eta }{r_{s}^{2}}\frac{3}{4\pi }\left(-\frac{1+2\eta +8\eta^{2}-5\eta^{3}}{3{\left(1-\eta \right)}^{3}}-\frac{\;\text{ln}(1-\eta )}{3\eta }\right), \end{array}$$

$$\begin{array}{c} \beta \kappa =\frac{\eta }{r_{s}}\frac{3}{4\pi }\left(\frac{4-10\eta +20\eta^{2}-8\eta^{3}}{3{\left(1-\eta \right)}^{3}}+\frac{4\;\text{ln}(1-\eta )}{3\eta }\right), \end{array}$$

$$\begin{array}{c} \beta \bar{\kappa }=\eta \frac{3}{4\pi }\left(\frac{-4+11\eta -13\eta^{2}+4\eta^{3}}{3{\left(1-\eta \right)}^{3}}-\frac{4\;\text{ln}(1-\eta )}{3\eta }\right). \end{array}$$

As we are only interested in relative difference of $F_{\mathrm{sol}}$, we set β=1 in the simulation.

The computationally intensive part of the simulation is the calculation of the geometric measures of a union of balls. Methods to efficiently compute these and also their derivatives have been developed in a computational topology context (22–26). The general approach of these methods can be described as follows. Take a union of balls $\underset{i}{⋃}B_{i}$ with centers $c_{i}$. Let $V=\underset{i}{⋃}V_{i}$ be the Voronoi tessellation defined by the $c_{i}$. The convex sets $V_{i}\cap B_{i}$ decompose the union of balls which is also known as the space filling diagram. The geometric measures are then calculated by inclusion–exclusion formulas of these convex sets and their intersections. To do this efficiently, the methods make use of the nerve of the decomposition, also known as the Alpha complex. The Alpha complex contains all incidence information of the sets $V_{i}\cap B_{i}$ and their intersections which correspond to the terms needed in the inclusion–exclusion formulas. These methods are implemented in the AlphaMol package (45). To avoid influences of a container on cluster formation, we simulated the particles in a box with periodic boundary conditions.

To analyze the configurations forming during the simulations in an automated way, we utilized the Graphs.jl package (46) to group clusters with isomorphic hard sphere contact graphs together. Given a configuration $x=(x_{1},...,x_{n})$, we define the hard sphere contact graph

$$\begin{array}{c} G_{\epsilon }\left(x\right)=\left(V\left(x\right),E_{\epsilon }\left(x\right)\right), \end{array}$$

with the set of nodes

$$\begin{array}{c} V(x)=\{i\in 1,\cdots ,n\}, \end{array}$$

and the corresponding edge set

$$\begin{array}{c} E_{\epsilon }\left(x\right)=\left\{\left\{i,j\right\}|i,j\in V\left(x\right),\;\text{dist}\left(x_{i},x_{j}\right)\leq 2R+\epsilon \right\}. \end{array}$$

As discussed before, $\epsilon =\frac{r_{s}}{2R}$ is the admissible error depending on the point in parameter space we consider.

## Supplementary Material

Appendix 01 (PDF)

Movie S1. Simulation run of eight hard spheres, which self assemble into a double helix configuration as a minimizing structure. The simulation is for a solvent density *η* of 0.475 and a solvent radius *r_s_* of 0.475.

## Data Availability

There are no data underlying this work.
