# Supplementary material for: Exotic self-assembly of hard spheres in a morphometric solvent
Source: Proc Natl Acad Sci U S A. 2024 Apr 4;121(15):e2314959121. doi: 10.1073/pnas.2314959121 (PMC11009619; doi:10.1073/pnas.2314959121)
Supplement: Supplementary file 1 — Appendix 01 (PDF) [file pnas.2314959121.sapp.pdf]

Supplementary Information for

Exotic self assembly of hard spheres in a morphometric solvent.

Ivan Spirandelli, Rhoslyn Coles, Gero Friesecke, Myfanwy E. Evans

Myfanwy E. Evans.
E-mail: evans@uni-potsdam.de

This PDF file includes:

Legend for Movie S1

Other supplementary materials for this manuscript include the following:

Movie S1

Movie S1. Simulation run of eight hard spheres, which self assemble into a double helix configuration as a minimizing structure. The simulation is for a solvent density η of 0.475 and a solvent radius r_s of 0.475.
